# The Arts as a Medium for Care and Self-Care in Dementia: Arguments and Evidence

**DOI:** 10.3390/ijerph15061151

**Published:** 2018-06-01

**Authors:** Justine Schneider

**Affiliations:** School of Sociology & Social Policy and Institute of Mental Health, University of Nottingham, Nottingham NG9 2HA, UK; Justine.Schneider@nottingham.ac.uk; Tel.: +44-115-846-7307

**Keywords:** dementia, arts, evidence, self-care

## Abstract

The growing prevalence of dementia, combined with an absence of effective pharmacological treatments, highlights the potential of psychosocial interventions to alleviate the effects of dementia and enhance quality of life. With reference to a manifesto from the researcher network Interdem, this paper shows how arts activities correspond to its definition of psycho-social care. It presents key dimensions that help to define different arts activities in this context, and illustrates the arts with reference to three major approaches that can be viewed online; visual art, music and dance. It goes on to discuss the features of each of these arts activities, and to present relevant evidence from systematic reviews on the arts in dementia in general. Developing the analysis into a template for differentiating arts interventions in dementia, the paper goes on to discuss implications for future research and for the uptake of the arts by people with dementia as a means to self-care.

## 1. Introduction

The field of dementia research has attracted a great deal of attention and investment in recent years. UK government funding for research concerning major causes of death (cancer, heart disease and dementia and stroke together) has been analysed to show that, between 2008 and 2012, the share allocated to dementia and stroke together grew 13% to 33% [1]. This upward trend exists in other countries with ageing populations; for example, the final budget of the Obama presidency in 2016 included a 60% increase in funding for dementia research, to $936 million, and in 2017 this had further increased to $1414 million [2]. Globally, most of the research effort has been focused on the search for causes and cures. However, the quest for a cure has proved disappointing so far, and some pharmaceutical companies have abandoned it altogether [3]. Rather than reversing the neurodegeneration that characterises dementia, drug development has turned to treating symptoms, and to early modification of the course of the disease, which may entail initiating treatment even before a person displays any symptoms of memory loss [4]. 

It follows that the population already living with dementia is not going to decline in the foreseeable future. Moreover, life expectancy continues to increase—and with it, the probability of developing a dementia. While general improvements to cardiovascular health in the UK appear also to benefit brain health and thus to have reduced the incidence of dementia, 210,000 persons per year are still expected to develop the symptoms of dementia [5]. In a Swedish epidemiological study in 2005–2007, 28% of individuals aged 85 had a dementia, rising to 45% at 95 [6]. In the absence of a cure, the growing number of people surviving with dementia—often for many years post-diagnosis, has galvanised pursuit of interventions to alleviate or delay its effects. Interventions that improve the functioning, quality of life and satisfaction of people with dementia are therefore in demand. 

A “manifesto”published in 2011 by Interdem—a European network of dementia researchers—highlighted the need for more research into psychosocial interventions, defined as relating to: “psychological and/or social functioning, including well-being and cognition, interpersonal relationships and everyday functional abilities, such as activities and daily living skills” [7]. The manifesto also states that the process or quality of the intervention is just as important as the outcomes it achieves. An acceptable intervention should promote: dignity, autonomy, reciprocity, lack of stigma and social integration. This paper sets out the case for adopting arts-based interventions to meet the psychosocial needs of people with dementia. 

The broad theme set out in this paper is that music, visual art and dance—taken here as representative art forms, have potential benefits to offer people with dementia, which include cognitive, emotional and social outcomes. In keeping with the manifesto, the arts also foster dignity, autonomy, reciprocity, lack of stigma and social integration. What follows is an attempt to elucidate these benefits. First, three dimensions of difference are explained, to help to define arts interventions for the purpose of evaluation and research. Then, examples of arts-based interventions taken from music, visual art and dance are described and illustrated, to acquaint the reader with current practice. The paper then puts forward five general arguments in favour of the arts in dementia, arguing that these constitute sufficient grounds for the arts to form part of everyday care, whether this is self-care or formal provision. Some research evidence concerning specific interventions is presented and appraised. Finally, needs for development and evaluation of arts-based intervention are discussed.

## 2. Defining and Differentiating Arts Interventions

It is challenging to define psychosocial interventions which operate by means of a recognised form or art. Cowl and Gaugler’s far-reaching systematic review [8] included in their search “visual arts (painting, coloring, and sculpting, or viewing works of art created by others), music (listening to, singing, and playing music), drama/movement (acting, storytelling, dance, and expressive movement), songwriting, and poetry (writing and reading of poetry).” (p. 284 [8]). Another way to categorise the arts is threefold, following Young et al. [9]: literary (e.g., reading aloud, poetry reciting, or creative writing); performing (e.g., music, dance, theatre) and visual (e.g., gallery visits, making art). It may be argued that cooking, gardening and needlework also qualify as arts, as do many other pursuits. However, little research has been undertaken in relation to manual arts and crafts in dementia. This paper therefore focuses on three forms of art where considerable bodies of evidence have been amassed: music, visual art, and dance, in descending order of research outputs. 

First, the activities that fall within each of these domains need to be understood with reference to some specific parameters. Like other psycho-social interventions, arts activities in dementia vary in fundamental respects. To understand the intervention process and to evaluate its impact, certain contextual factors also need to be specified. These will affect which outcomes are relevant and feasible to evaluate. Three contextual factors will be considered here: the level at which the intervention is delivered, the severity of dementia in the person participating, and who is delivering the activity. These are not the only dimensions on which we may compare different arts interventions; questions of place and space may be relevant, as well as differences in the intentions and outcomes of the activity, and the involvement of close persons and care partners. 

### 2.1. Intervention Level

Some interventions target the individual, some the caring relationship (formal or informal) and some the way that care is provided. For instance, among the range of psychosocial interventions directed at the individual with dementia are light therapy [10], aromatherapy [11] and cognitive therapy [12]. Approaches designed to improve the caring relationship include: training in dementia care mapping [13], communication skills training [14,15], and informal-carer support interventions [16]. To influence the way that care is organised, interventions are even more complex and often involve several components; examples are care management [17] or integrated care [18]. Multi-level programmes such as Therapeutic Thematic Arts Programming (TTAP) [19] may be included in this organisational category because they are intended to act on all three levels; the individual, carer and organisation providing care. 

### 2.2. Severity of Dementia

In people with dementia, it is also relevant to consider for what level of severity or symptoms a given arts intervention may be suitable. Skills in activities of daily living are impaired as the disorder advances [20]. Cognition declines as dementia progresses over time [21], with the result that functional impairment may affect a person’s ability to engage with different arts interventions. Consequently, for instance, dance interventions for people with dementia often rely on seated movement, to avoid the risk of falls in those whose balance may be impaired. Other features of activities suited to a population with more advanced dementia are multi-sensory experiences of colour, touch and sound, usually mediated by close human contact, e.g., [22,23]. Outcomes also need to be selected according to the severity of participants’ dementia. Numerous intervention studies have been implemented with people with advanced dementia where adverse symptoms such as agitation or depression are targeted e.g., [10,11]. Reviews indicate that there is evidence to support use of music therapy [23], occupational activities and sensory intervention to directly help people with dementia who suffer agitation [24]. 

### 2.3. Facilitator

Who delivers the intervention is a further variable, which has a bearing on quality: is the facilitator trained in participatory arts, what is their knowledge of dementia, how much experience do they bring to a particular session? The therapeutic arts professions each have their own discipline and body of evidence, within a common psychoanalytical tradition, with professional training, qualifications and governing bodies. Applied to people with dementia, art therapy [22], music therapy [23] dance [25] and drama therapy [26] therefore need to be seen as comprising a separate category beyond the scope of this paper. However, it should be noted that therapeutic arts professionals in the field of dementia are relatively rare. For example, of approximately 1000 qualified music therapists in the UK, fewer than one in four works with people with dementia, so capacity to meet the needs of this growing number of potential beneficiaries is severely limited [27]. The highly-skilled provision offered by therapeutic arts professionals is available from private practitioners to those people with dementia who can afford to pay; in other words, the majority of people with dementia who live in the community are not likely to obtain access to this specialist kind of arts intervention. 

The arts may be used to entertain or to distract, as well as to comfort, stimulate, soothe or affirm the identity of a person with dementia. In order to understand any arts intervention, the perspective of the person delivering it should be considered. Is this person guided by a particular theory or purpose? What are their qualifications and experience? A professionally-trained artist may not be familiar with people with dementia, while an activity co-ordinator with experience of dementia may have only a rudimentary understanding of the art they are facilitating. In the following sections three well-recognised approaches—visual art, music and dance—are discussed.

### 2.4. Visual Art

Visual art is taken here to include gallery visits to immerse people in the work of other artists, but also painting, making sculptures and handling objects. The seminal art appreciation programme for people with dementia began at New York’s Museum of Modern Art [28,29]. Art gallery visits with discussion and a facilitative guide are a form of intervention that has been adopted in different countries, including Australia [30], the UK [31], The Netherlands [32] and Switzerland [33]. In some places, viewing art has been combined with making art [34]. These interventions typically operate at the level of the individual with dementia and the individual-care partner dyad. 

The English Longitudinal Study of Ageing shows that for the general population growing older is associated with increasing involvement in activities such as going to museums, theatre or cinema [35]. If visiting museums is normal for older people, then these forms of cultural engagement may be an indicator of social inclusion for people with a diagnosis of dementia. The presence in public spaces of people with dementia presents a challenge to fear and prejudice, and may help to dispel stigma. The Alzheimer’s Society UK have published a guide to dementia-friendly arts venues which offers advice on preventing and overcoming barriers to cultural inclusion faced by people with dementia. This gives arts providers some responsibility for promoting the integration of people with dementia. The benefits that follow arguably accrue to the whole of the community by making society more diverse. In addition, for individuals who cannot be included culturally by any other means, and for people who prefer to remain in their own homes, computer applications have been developed to give access to art collections online. These are under evaluation for people with dementia [36]. 

### 2.5. Music

Music interventions encompass listening, playing instruments and singing. The Interdem manifesto highlights the qualitative superiority of certain outcomes for people with dementia, including a “sense of purpose” and “pleasure”. There are striking examples of instances where the medium of art provides access to an otherwise-unreachable personal history and identity. For instance, more than two million people have viewed the rough cut from the film *Alive Inside*, which documents the introduction of playlists containing preferred music to people whose lives are limited by dementia and other disabilities. The video *Man In Nursing Home Reacts To Hearing Music From His Era* [37] illustrates a transformation through engagement with music by a man who was previously disengaged. The delight that the music brings to him is infectious. This not only changes the mood of the person with (presumed) dementia; it alters our perceptions as onlookers. It also clearly has a positive effect on the relationships between that man and his family as well as between him and his paid carers. Witnessing this change in her charge’s demeanour brings a tear to the eye of the nursing assistant, raising the possibility that the encounter with music has generated empathy for his condition. Communication between carers and individuals with dementia by means of art—in this case, music, appears to aid reassertion of the individual’s identity and others’ recognition of personhood, and may therefore lead to greater respect and sensitivity in all concerned. Whether this has further benefits in terms of care quality is a question that remains to be addressed. Nevertheless, supporting the person with dementia to access preferred music appears to have improved his psychological and social functioning, increased well-being and communication, and revived interpersonal relationships, at least in the short term. Just as computer applications have extended the possibilities for accessing art, the use of personal digital playlists has grown, offering another example of how technology can be harnessed to promote arts interventions in dementia.

### 2.6. Dance

Dance may include movement that is accompanied by music or rhythm, such as chair-based exercises, as well as performance by people with dementia. Choreographer Filipa Pereira Stubbs produced *Smile*, an ensemble performance that includes people with dementia who regularly attend dance sessions [38]. The video notes state that dance moves us away from the boundaries that living with dementia can impose. It restores a sense of community, of supporting one another, of being important to one another. This points to the manifesto criteria of reciprocity and social integration. In the *Smile* video, we see people with dementia as co-creators of a beautiful work of art, absorbed in purposeful activity and radiating pleasure. A viewing of this performance shows individuals improvising movement to the music (autonomy) in a public performance, where the dignity of participants is enhanced by their presence—literally and metaphorically, in the spotlight. In short, *Smile* speaks to all the benchmarks of quality arts interventions listed in the Interdem manifesto; dignity, autonomy and reciprocity. 

Despite a strong theoretical foundation [39,40] and work to apply this to dementia care [41,42,43], the evidence concerning dance in dementia does not have a high profile. This may be due to the time lag between publication and practice developments, but it may also be due to a shortage of practitioners in the field. In her review, when considering the dance-movement therapy literature, Beard [44] suggests that non-verbal communication is a primary benefit, citing Nystrom and Lauritzen [45], who argue that movement can be used to substitute for speech, to express thoughts, memories, and emotions. Showing people with dementia as capable of communicative actions emphasises how meaning is jointly-constructed (p. 642 [44]). We co-create meaning consciously when we participate in the arts, but this is also a feature of everyday life. 

## 3. Discussion: A Rationale for the Arts in Dementia Care and Self-Care

Creativity is not dependent on memory. That is the message that Ann Basting promulgates through her book *Forget Memory* [46] and her TEDx Talk [47]. These and other testimonies illustrate that creative processes are used to evoke memories [48], enable people to communicate about their experience [9], to reinforce identity [49], and to strengthen relationships with their own family members [50], as well as with care personnel and others involved in the intervention process [51].

For readers whose interests lie principally in dementia care, dementia self-care, rehabilitation or “living well with dementia”, the case for the arts as a change mechanism is compelling. Their benefits are consistent with the principles of compassion and of relational (person-centred) care that guide contemporary practice [52]. From a pragmatic perspective, the arts present the possibility of lower costs than interventions delivered in a clinical context, for a number of reasons. Unlike the therapeutic professions, in the UK community arts practitioners are not a scarce commodity. Most have relatively low overheads compared to, say, a clinical psychologist, who depends on the health service infrastructure to deliver care, or an occupational therapist who works within a local authority system. Of course such organisational structures may provide governance and some quality assurance of the services provided, whereas independent arts practitioners are not regulated. The arts are non-invasive, in research terms, and risks presented by arts activities for people with dementia are not very different from those they meet in everyday activities, such as fatigue, trips and falls or accidental ingestion of something that is not food. 

Leaving aside questions about effectiveness, the discussion so far highlights five good reasons to support the arts for people with dementia:

1. People with dementia usually enjoy participating in art, whether actively creating art or as an appreciative audience.
2. The arts can remain accessible despite memory loss because of their multi-sensory nature and the possibility of experiencing art in the moment irrespective of prior knowledge or associations.
3. Carers—both professional and family supporters—get double benefit from arts interventions; their own enjoyment as participants, and that of seeing their charges enlivened or soothed.
4. The wider community benefits from the fostering of cultural capital in any segment of society, including older people with dementia.
5. Art does little harm, indeed it often fosters social interaction and a sense of belonging.

In themselves, and without further empirical evidence, these five points provide a strong rationale for promoting the arts in dementia. They offer sufficient grounds for transforming activities in day centres and residential care to include tried and tested artistic activities such as those illustrated here, or to adopt an integrated programme using different media such as TTAP [19]. The obstacles to improving care through the arts are less likely to be financial than to constitute resistance to change from a workforce whose comfort zone lies in giving manicures and playing bingo. However, in addition to rational argument, a growing body of evidence supports the use of the arts in dementia.

### 3.1. Art Presents Challenges to Research Methodology

Measurement may seem irrelevant because the experience of art changes us without much use of our rational faculties. Even without a measure of effect size, viewing a piece like *Smile* or *Alive Inside* influences our expectations and aspirations for life with dementia more powerfully than an academic treatise or peer-reviewed paper. Ann Basting speaks passionately about the arts as a way of being in relationship [53]. One challenge for researchers is to know where they are located within that relationship—and how this creates a methodological problem for their enquiry [54]. Another is the huge diversity of artistic forms and activities, making it difficult to replicate the arts as interventions consistently and reliably.

Nonetheless, there is ample innovation and proof-of-principle evidence to make the arts a fertile field for dementia researchers in pursuit of evidence. Meta-reviews of psychosocial interventions are beginning to place arts interventions on a par with other non-pharmacological approaches to alleviate certain behavioural symptoms of dementia, such as agitation and apathy [55,56]. One of the broadest systematic reviews of the arts in dementia, cited earlier in this paper [8], summarises the characteristics of the included studies, and this gives an overview of the contemporary research field (Table 1). 

Cowl and Gaugler looked in detail at the 63 studies with quantitative results. Most studies looked at agitation, apathy, cognition, depression and mood as outcomes. According to Beard [44], the predominance of biomedical outcomes such as these demonstrates a bias away from person-centred, in-the-moment benefits and in favour of the needs of carers (pp. 639–640 [44]). None of the studies reviewed by Cowl and Gaugler found an effect on cognition but at least one of the measured outcomes showed significant improvement in 46 (73%) of these studies, as described below. The distribution of research across arts modalities is uneven, even in relation to the three topics selected here, with music and visual art receiving the most attention and relatively little effectiveness evaluation in the field of dance. In the way that new studies build on old, the disparity is likely to continue if not increase. 

There were 14 randomised controlled trials (RCTs) identified by Cowl and Gaugler. One was not peer-reviewed and did not report the sample size. Eleven of the 13 remaining RCTs studies used music as the mode of interventions, the other two used visual art. Eleven studies showed improvement (statistically significant at 5% level). There were positive effects on mood, alleviating depression and apathy from art therapy and colouring pictures [57,58]. Similar outcomes were associated with the use of music [59,60]. Music appeared to improve behaviour and increase sociability [60,61,62,63,64], as did visual art practice in one multi-site study [57]. Visual art interventions were associated with greater wellbeing and quality of life [57,58], while music interventions were associated with better language functioning [65,66].

The Cowl and Gaugler review does not differentiate interventions within each art form according to the dimensions set out in this paper: the level of intervention, the severity of dementia, and the qualifications of the facilitator, and this makes it difficult to apply the findings in practice. Subsequent reviews of the arts in dementia sometimes also fail in this respect and therefore add only limited information, frequently confounding evidence from the therapeutic professions with evidence concerning arts facilitated by people with different skills and objectives, which is indicated by the indiscriminate use of the terms “art therapy” or “music therapy”. This flaw in the earlier Cochrane review of music interventions in dementia has been corrected in the latest review [66].

### 3.2. Comparing Arts Interventions

This brief stocktake of arts interventions used with people with dementia indicates further questions that need to be addressed to understand the effects of the arts on people with dementia:

- For what purpose? Is the arts activity an end in itself or a means to change of some kind? This might include influencing the general public e.g., de-stigmatisation.
- For each level at which the intervention operated, what are the outcomes of interest?
- What is the role of the person with dementia? If actively participating, how much and with what support?
- Who else is involved and how?

Incorporating these questions to the variables of intervention level, severity of dementia and facilitator discussed earlier, Table 2 attempts to apply the resulting list of seven dimensions to the three examples of interventions described above; the gallery visits to the Metropolitan Museum of Art (MOMA), the playlist intervention portrayed in *Alive Inside*, and the dance performance *Smile*.

## 4. Conclusions

There is a growing recognition that the arts can make a contribution to the quality of life of people living with dementia. This impact can be direct, by bringing emotional release or pleasure to the person affected. It can also be indirect, by giving carers a stronger sense of purpose, resolve and commitment, or by making the wider community more compassionate and aware. Art as an aesthetic experience in the moment may be sufficient justification for its promotion for people with dementia, and for its use in self-care. In addition the arts can clearly have instrumental, therapeutic effects on people with dementia, and this is where the greatest number of empirical studies focus [67]. With relatively low costs and minimal risks, there remain promising areas for further evaluation and testing of arts interventions. The analysis of seven dimensions on which these may differ is given here to facilitate further research. The modalities to be prioritised in this research should undoubtedly include dance and movement, which has been neglected despite its strong empirical tradition. 

The arts afford a means to self-care in dementia that has not been widely promoted. While a diagnosis of dementia is an unwelcome harbinger of disability and decline that is likely to be more accelerated than ageing without dementia, it should not exclude anyone from their customary cultural activities, nor from engaging in new ones. There is a growing recognition that people with dementia, like all disabled people, are entitled to all the rights of citizenship. A social understanding of disability highlights the responsibility of the wider society to ensure that these entitlements are realised. People affected by dementia should be encouraged to assert their rights to cultural inclusion. In anticipation of cognitive and functional decline, it would be advisable to explore a range of expressive modalities through the arts, knowing that these activities may well extend one’s capacity to communicate despite dementia. In fact, there is no need to wait for a diagnosis of dementia to embark on artistic exploration or to extend one’s artistic practice. This is something that anyone can do who is concerned about the possibility of developing dementia. The arts afford enriching experiences to all of us, with or without a diagnosis of dementia.

## Figures and Tables

**Table 1:** After Cowl and Gaugler (2014), p. 286 [8].

Aspect	Results of Review
Intervention	39 visual art, 53 music, 3 drama (1 including dance), 2 poetry, 15 combination
Study design	49 case studies, 46 quasi-experimental studies, 14 randomised controlled trials, 3 descriptive studies
Methodology	50 quantitative, 49 qualitative, 13 both
Number of participants	1699 people with dementia, 403 paid carers, 94 care partners
Duration of intervention	Ranged from 10 minutes to 3 years
Length of follow-up	Ranged from none to 24 weeks

**Table 2:** Characterising three arts interventions on seven dimensions.

	Name	MOMA *	Alive Inside	Smile
	Modality	Viewing art and discussing it in a group	Listening to music	Dance and movement
1	What is the purpose?	To generate group discussion, emotional responses, associations, cognitive activity	To improve mood and social engagement	To develop and demonstrate skills, co-create, perform, communicate
2	At what level is change intended?	Individual and caring relationship	Individual and caring relationship	Individual and (potentially) caring relationship
3	People with how much impairment are shown to benefit?	Mild to moderate	High	Mild to moderate
4	What outcomes are apparent in the video?	Pleasure Contemplation	Pleasure Vocalisation Animation	Pleasure Activation Bodily expression
5	What is the role of the person with dementia?	Active participant in a process designed to be intellectually and emotionally stimulating	Passive recipient of technologically-based intervention	Active participant in physically and intellectually demanding exercise
6	Who is delivering the activity?	A fine art expert, probably withpreparation regarding dementia	A social worker or similar support worker or volunteer	A dance professional with extensive experience, aided by other dancers
7	Who else is involved and how?	Carers and other people with dementia	Carers and family members	Dancers and other people with dementia

* Metropolitan Museum of Art.
